# Assessing *Symbiodinium* diversity in scleractinian corals via next-generation sequencing-based genotyping of the ITS2 rDNA region

**DOI:** 10.1111/mec.12869

**Published:** 2014-08-18

**Authors:** Chatchanit Arif, Camille Daniels, Till Bayer, Eulalia Banguera-Hinestroza, Adrian Barbrook, Christopher J Howe, Todd C LaJeunesse, Christian R Voolstra

**Affiliations:** * Red Sea Research Center, King Abdullah University of Science and Technology (KAUST)23955, Thuwal, Saudi Arabia; † GEOMAR Helmholtz Centre for Ocean Research Kiel24105, Kiel, Germany; ‡ Department of Biochemistry, University of CambridgeBuilding O, Downing Site, Tennis Court Road, Cambridge, CB2 1QW, UK; § Department of Biology, Penn State UniversityUniversity Park, PA, 16802, USA

**Keywords:** coral reefs, dinoflagellates, internal transcribed spacer 2, next-generation sequencing, operational taxonomic unit, *Symbiodinium*

## Abstract

The persistence of coral reef ecosystems relies on the symbiotic relationship between scleractinian corals and intracellular, photosynthetic dinoflagellates in the genus *Symbiodinium*. Genetic evidence indicates that these symbionts are biologically diverse and exhibit discrete patterns of environmental and host distribution. This makes the assessment of *Symbiodinium* diversity critical to understanding the symbiosis ecology of corals. Here, we applied pyrosequencing to the elucidation of *Symbiodinium* diversity via analysis of the internal transcribed spacer 2 (ITS2) region, a multicopy genetic marker commonly used to analyse *Symbiodinium* diversity. Replicated data generated from isoclonal *Symbiodinium* cultures showed that all genomes contained numerous, yet mostly rare, ITS2 sequence variants. Pyrosequencing data were consistent with more traditional denaturing gradient gel electrophoresis (DGGE) approaches to the screening of ITS2 PCR amplifications, where the most common sequences appeared as the most intense bands. Further, we developed an operational taxonomic unit (OTU)-based pipeline for *Symbiodinium* ITS2 diversity typing to provisionally resolve ecologically discrete entities from intragenomic variation. A genetic distance cut-off of 0.03 collapsed intragenomic ITS2 variants of isoclonal cultures into single OTUs. When applied to the analysis of field-collected coral samples, our analyses confirm that much of the commonly observed *Symbiodinium*ITS2 diversity can be attributed to intragenomic variation. We conclude that by analysing *Symbiodinium* populations in an OTU-based framework, we can improve objectivity, comparability and simplicity when assessing ITS2 diversity in field-based studies.

## Introduction

Coral reef communities depend critically on the relationship between scleractinian corals and photosynthetic endosymbionts in the genus *Symbiodinium*. While the coral host provides a light-rich, sheltered environment and inorganic nutrients, the dinoflagellate algae provide photosynthetically fixed carbon (Muscatine & Cernichiari 1969). Some corals seem to rely strictly on a distinct symbiont type, whereas others harbour different types of *Symbiodinium* (Rowan & Knowlton 1995). Although host identity is the primary predictor of symbiont identity, coral-associated *Symbiodinium* diversity also covaries with geographical location, water depth and health state (Rodriguez-Lanetty *et al.* 2001; Toller *et al.* 2001; LaJeunesse *et al.* 2004, 2010; Sampayo *et al.* 2007; Macdonald *et al.* 2008; Finney *et al.* 2010; Tonk *et al.* 2013b). The current decline in coral reef cover resulting from global (e.g. ocean warming) and local (e.g. pollution, overfishing) anthropogenic factors has intensified the need to examine the spatial, geographical and ecological distribution of *Symbiodinium*–coral associations and how these relate to environmental extremes.

Various DNA markers have been used to describe genetic diversity within *Symbiodinium* (Sampayo *et al.* 2009). Rowan & Powers (1991) were the first to examine *Symbiodinium* evolutionary relationships using restriction fragment length polymorphisms (RFLPs) of the small ribosomal subunit RNA (SSU) gene from dinoflagellates from various marine invertebrate hosts. Their phylogeny identified divergent lineages within this genus, which exhibited sequence differences comparable to those observed among dinoflagellates from different taxonomic families and orders (Rowan & Powers 1992). These lineages are commonly referred to as clades. So far, nine clades are identified (clades A–I), of which representatives in six clades (i.e. A, B, C, D, F and G) have been shown to persist in association with corals, other cnidarians, giant clams and sponges (Baker 2003; Pochon *et al.* 2006). It was soon recognized that the conserved SSU gene is not able to resolve species (Rowan & Powers 1991; McNally *et al.* 1994; LaJeunesse 2001). Researchers turned to more variable DNA regions, including the nuclear large subunit (LSU) (Wilcox 1998; Loh *et al.* 2001), the internal transcribed spacer regions (Hunter *et al.* 1997; LaJeunesse 2001; van Oppen *et al.* 2001), the chloroplast large subunit (cp23S) (Santos *et al.* 2002; Pochon *et al.* 2006) and cytochrome oxidase b (Sampayo *et al.* 2009). A systematic survey by Sampayo *et al*. (2009) targeting ribosomal, mitochondrial and chloroplast genes employing 13 distinct genetic analyses found that different markers showed remarkable concordance, but differed in their relative ability to resolve ecologically distinct units. Other rapidly evolving markers, including the chloroplast *psbA* noncoding region (Moore *et al.* 2003; LaJeunesse & Thornhill 2011) and microsatellites, were recently employed, often in combination with each other, to improve genetic resolution further and delimit species boundaries (Thornhill *et al.* 2014). Only recently, Barbrook *et al*. (2014) sequenced the entire chloroplast genome of a *Symbiodinium* sp. type *C3*, where individual genes reside on minicircles that will provide additional genetic markers. Currently, however, the ITS2 region is still the most commonly employed DNA marker used to assess *Symbiodinium* diversity from a diverse array of hosts and over large geographical distances (LaJeunesse *et al.* 2010; Wicks *et al.* 2010; Silverstein *et al.* 2011; Putnam *et al.* 2012; Tonk *et al.* 2013a).

LaJeunesse (2002) determined that ITS2, when analysed through targeting of numerically dominant intragenomic variants, provides sufficient resolution to resolve many ecologically distinct *Symbiodinium* spp. To achieve this, denaturing gradient gel electrophoresis (DGGE) was used to screen PCR amplifications for common ITS2 sequence variants diagnostic of a particular *Symbiodinium* ‘type’ (LaJeunesse 2002; Sampayo *et al.* 2007; LaJeunesse *et al.* 2010; Silverstein *et al.* 2011). More recently, researchers have also employed bacterial cloning and sequencing and found more sequence diversity within the populations of *Symbiodinium* residing in a host than identified with DGGE (Apprill & Gates 2007; Stat *et al.* 2009, 2011, 2013). Because rDNA represents an extreme example of a multicopy gene arrayed in tandem, intragenomic variation in the form of pseudogenes or numerous low-abundant functional variants affect how ITS2 data are interpreted (Thornhill *et al.* 2007; Sampayo *et al.* 2009; LaJeunesse & Thornhill 2011). For instance, it has been shown that cloning-and-sequencing-based approaches are potentially prone to inflated diversity estimates through the technique's tendency to recover intragenomic variants that are of low abundance (i.e. limited diagnostic value), as well as by introducing additional sequence artefacts generated during the PCR and cloning steps (Thornhill *et al.* 2007). However, sequencing a substantial number of clones may resolve how rDNA data are best analysed and interpreted by allowing differentiation between intragenomic and interspecific ITS2 sequence variants, albeit at a high cost (Sampayo *et al.* 2009). Assessment has also shown that individuals from different species possess widely differing amounts of intragenomic variation (Thornhill *et al.* 2007), yet the full extent of this variation is at present unknown. In both regards, the application of pyrosequencing-based methods is projected to overcome the limited resolution and high cost associated with Sanger-based ITS2 diversity assessments, as a high number of sequences are produced at comparatively low cost per sequence.

While the study of prokaryotic diversity is now routinely conducted via pyrosequencing-based amplicon typing of the 16S ribosomal RNA gene (Sogin *et al.* 2006; Roesch *et al.* 2007), only a limited number of studies have utilized high-throughput sequencing to assess eukaryotic diversity. For instance, Stoeck *et al*. (2010, 2009) and Amaral-Zettler *et al*. (2009) sequenced the variable regions of the small subunit (SSU) and large subunit (LSU) of the 18S region to estimate eukaryotic diversity.

Pyrosequencing is now being applied to assess *Symbiodinium* diversity (Green *et al.* 2014; Quigley *et al.* 2014; Thomas *et al.* 2014), but there is a need to ground truth this new approach. In this study, we applied pyrosequencing of ITS2 rDNA amplicons (320–360 bp) to genotype *Symbiodinium* diversity in several isoclonal and replicated cultures, two cultured isolates mixed at different ratios and field-collected coral specimens. The application of a high-throughput pyrosequencing approach holds the promise to improve assessment of the relative degree of sequence variation found in these eukaryotic genomes. Further, a pyrosequencing approach may provide a more accurate detection of low-abundance background *Symbiodinium*. Our aim was to understand diversity of the ITS2 gene at the genome level and to compare pyrosequencing results to data obtained from DGGE typing. Furthermore, we sought to analyse *Symbiodinium* diversity in an OTU-based framework in order to improve objectivity, comparability and simplicity when assessing ITS2 to study *Symbiodinium* composition in environmental samples of corals.

## Materials and methods

### Sample collection and processing

Isoclonal cultures of *Symbiodinium* sp. (CCMP2467: *Symbiodinium microadriaticum*, KB8: *S. microadriaticum*, rt-147: undescribed clade B type and rt-064: undescribed B1 type) were cultured at 23 °C in f/2 medium (Guillard & Ryther 1962) on a 12 h/12 h light–dark cycle (daytime: 6 am to 6 pm; night-time: 6 pm to 6 am, light intensity 80 μmol/m^2^/s). The salt content in the medium was set to 40 g/L, matching the average salinity characteristic of the Red Sea. Coral samples were collected from various reefs in the Red Sea between 2011 and 2012 with SCUBA at depths between 4 and 12 m. More specifically, five specimens of *Pocillopora verrucosa* were collected from reefs at Maqna (*n* = 2), Al Wajh (*n* = 2) and Doga (*n* = 1) as well as one specimen of *Acropora hemprichii* from Al Fahal reef at Thuwal. Specimens of about 1–3 cm^2^ tissue were collected with hammer and chisel and stored in Whirl-Paks during diving, subsequently washed with 0.22-μm-filtered sea water (FSW), preserved in DMSO/NaCl buffer (Gaither *et al.* 2010) and stored at 4 °C until further processing. For *Symbiodinium* culture DNA extraction, cells from each culture were counted using a haemocytometer (Hausser Scientific, Horsham, PA) under a light microscope (Leica DM2500, Wetzlar, Germany), and ∼8 × 10^6^ cells per culture were used for DNA extraction. Mixed samples were generated by combining cells of two *Symbiodinium* species (a strain of *S. microadriaticum* CCMP2467 from clade A and strain rt-147 from clade B) in a ratio of 1:1 (4 × 10^6^ cells: 4 × 10^6^ cells) and in a ratio of 1:3 (2 × 10^6^ cells: 6 × 10^6^ cells), respectively. Cells were spun at 1934 g for 10 min and subsequently washed with DNase-free water. Five hundred microlitres of 0.5-mm sterile glass beads (BioSpec, Bartlesville, OK) was added to the pelleted cells together with 400 μL of buffer AP1 and 4 μL RNAse (Qiagen, Hilden, Germany). Samples were bead-beaten for 90 s with a Tissue Lyser II (Qiagen). DNA was isolated with the Qiagen DNeasy Plant Mini Kit (Qiagen) according to the manufacturer's instructions. For the analysis of technical variation, DNA from isoclonal cultures of CCMP2467 and rt-147 was isolated once but amplified in distinct PCRs with a different barcoded primer. For DNA extraction of environmental samples, ∼50 mg of coral tissue was transferred to 1.5-mL tubes (Eppendorf, Hamburg, Germany). Five hundred microlitres of 0.5-mm sterile glass beads (BioSpec) was added together with 400 μL of buffer AP1 and 4 μL RNAse (Qiagen). Samples were bead-beaten for 90 s with a Tissue Lyser II (Qiagen). DNA was isolated with the Qiagen DNeasy Plant Mini Kit (Qiagen) according to the manufacturer's protocol.

### DGGE analysis of Symbiodinium ITS2 rDNA

DGGE ITS2 diversity typing was performed following the protocol detailed in LaJeunesse (2002). Briefly, the *Symbiodinium* ITS2 region was amplified with the primer pair ITSintfor2 and ITS2CLAMP using PCR conditions described in LaJeunesse *et al*. (2003) with the following modifications: the annealing temperature was maintained at 52 °C for 27 cycles after 20 cycles of touchdown amplification. PCR products were mixed with 10 μL Ficoll-based loading buffer and concentrated by a speed vacuum before loading on an 8% polyacrylamide gel using a Cipher DGGE kit (CBS Scientific Company, Del Mar, CA). Gels were run at 150 V for 15 h, stained for 30 min with 1× SYBR Green (Invitrogen, Carlsbad, CA) and visualized on a Dark Reader Transilluminator (Clare Chemical Research, Dolores, CO). Prominent band(s) were excised from the DGGE gel with a sterile scalpel. Each band was transferred into an Eppendorf tube that contained 500 μL DNase-free water and incubated at 4 °C for 24 h. Two microlitres of this were used for reamplification as described in LaJeunesse (2002) and purified with Illustra ExoStar (SelectScience, Bath, UK) enzyme mix following the manufacturer's instructions. Successful amplification was verified by running products on a 1% agarose gel stained with 1× SYBR Safe (Invitrogen). Samples were sent for bidirectional Sanger sequencing at the KAUST BioScience Core Laboratory (Thuwal, Saudi Arabia). Sequences were processed in CodonCode Aligner (CodonCode Corporation, Centerville, MA). After quality trimming, forward and reverse sequences were assembled into contigs. For phylogenetic assignment of ITS2 sequences, we built a custom BLAST database (File S1, Supporting information) of ITS2 types collected from 409 ITS2 sequences taken from GeoSymbio (Franklin *et al.* 2012) (denoted as GS), 7 ITS2 sequences from Scott Santos’ database (www.auburn.edu/∼santosr/sequencedatasets.htm) (denoted as ST) and 17 DGGE ITS2 sequences from Todd LaJeunesse's SD2-GED database (https://131.204.120.103/srsantos/symbiodinium/sd2_ged/database/views.php) denoted as LJ. ITS2 sequences were assigned to the ITS2 types that represented highest identity in the BLASTN hits.

### 454 pyrosequencing of Symbiodinium ITS2 rDNA

PCR amplification of the ITS2 gene for pyrosequencing was performed using primers ITSintfor2 and ITS2-reverse that generated an amplicon of around 320 bp. The primer sequences were 5′- CCATCTCATCCCTGCGTGTCTCCGACTCAG(N)_8_GAATTGCAGAACTCCGTG-3′ (454-ITSintfor2) and 5′- CCTATCCCCTGTGTGCCTTGGCAGTCTCAGGGGATCCATATGCTTAAGTTCAGCGGGT-3′ (454-ITS2-reverse). Primers included 454 Lib-L library adapters (underlined) and a barcode (shown as N) (Hamady *et al.* 2008). PCRs were run in triplicate per sample with 12.5 μL of Qiagen Multiplex PCR Kit (Qiagen), 0.1 μm primers, 20–50 ng DNA and DNase-free water to make a total volume of 25 μL. The following PCR conditions were used: initial denaturation for 15 min at 94 °C, followed by 35 cycles of 94 °C for 30 s, 51 °C for 30 s, 72 °C for 30 s and a final extension step of 10 min at 72 °C. PCR products were run on a 1% agarose gel stained with 1× SYBR Safe (Invitrogen) to visualize successful amplification. For each sample, triplicate PCR products were pooled, and their DNA concentrations were measured using a Qubit 2.0 (Invitrogen). Twenty nanograms of the triplicated PCR samples from all specimens was combined and ran on a 1% agarose gel to remove excess primers. The gel band was excised, purified with the Qiagen MinElute Gel Extraction Kit (Qiagen) according to the manufacturer's instructions, quantified with Qubit 2.0 (Invitrogen) and quality checked via Bioanalyzer (Agilent, Santa Clara, CA). Forty nanograms of this pooled library (i.e. pooled triplicated PCRs from all samples) was submitted to KAUST BioScience Core Laboratory for sequencing using Titanium FLX chemistry on a quarter of a picotiter plate. Raw sequencing data were retrieved with Roche 454 amplicon-processing pipeline.

### 454 pyrosequencing data analysis

A total of 218 475 reads with a median length of 314 bp were obtained from sequencing and processed using the software mothur version.1.31.2 (Schloss *et al.* 2009). Sequences were denoised using PyroNoise (Quince *et al.* 2011). Forward primer and barcode sequence were removed from reads by the trim.seqs function in mothur. All sequences that met the following criteria were discarded: barcodes (>0 mismatches), forward primer (>2 mismatches), ambiguities (>0 bp), homopolymers (>4 bp) and short sequence length (<250 bp). cutadapt version 1.1 (Martin 2011) was applied to remove the reverse primer (overall error rate set to 0.15). All identical sequences were subsequently collapsed, and representative sequences were retained via unique.seqs command in mothur. After chimera removal with UCHIME as implemented in mothur (Edgar *et al.* 2011), singletons (i.e. sequences detected only once across the entire data set) were also discarded. A total of 197 181 sequences were retained for the remainder of the analyses. From these data, frequency distributions of ITS2 variants for all samples were obtained via count.seqs command in mothur. Genetic distances for within-culture ITS2 diversity were calculated with the dist.seqs command in mothur based on MUSCLE-aligned ITS2 copies that were represented by at least 100 reads in any given isoclonal culture.

For the OTU-based framework analysis, ITS2 sequences were assorted into their respective clades based on pairwise distances via pairwise.seqs command, and subsequent clustering with average neighbour algorithm using a cut-off value of 0.15 (empirically determined to effectively cluster sequences into clades). Sequences assigned to distinct clades were aligned using MUSCLE (Edgar 2004) and trimmed to equal length using the screen.seqs and filter.seqs commands in mothur. Sequences that were shorter than 90% of sequences in each clade were discarded, resulting in 197 128 sequences. A distance matrix was calculated using the aligned sequences within each clade. Sequences were clustered with the average neighbour algorithm (Schloss & Westcott 2011) at a 97% similarity cut-off, as this cut-off clustered ITS2 variants from isoclonal cultures into a single OTU (i.e. species). The most abundant sequence in each OTU was chosen as the representative ITS2 copy and was annotated via BLASTN against the custom ITS2 database to determine a specific *Symbiodinium* type. In addition, the most abundant ITS2 copy from each pyrosequenced sample was aligned to the sequence derived from the most prominent DGGE band of the same sample to verify whether they were identical. A list of succession of commands and an unattended script are available as supplementary information (Files S2 and S3).

### Single-cell PCRs on Symbiodinium cells

To conduct single-cell PCRs on *Symbiodinium*, we developed an approach modified from Frommlet & Iglesias-Rodríguez (2008). Cells from cultures CCMP2467, KB8, rt-064 and rt-147 were harvested with the following changes: 1 mL of each culture was filtered through a 40-μm cell strainer (BD Biosciences, San Jose, CA), spun down at 1934 g for 10 min and washed 3 times with 1 mL of 1× TE buffer. Cell pellets were subsequently resuspended with 500 μl of 1× TE, counted using a haemocytometer (Hausser Scientific) and diluted to a concentration of 1 cell/μL (‘dilution-to-extinction’ approach). One microlitre was subsequently transferred into wells of 96-well plates, and the presence of single cells was confirmed using an inverted microscope (Leica DMI3000B). Cells were disrupted by triplicate freezing of cells for 2 min in liquid nitrogen and immediate thawing for 2 min at 95 °C. Next, a single 0.5-mm glass bead (BioSpec) was added to each well, and the cell was homogenized with a Tissue-Lyzer II (Qiagen) for 1 min. PCR master mix containing 12.5 μL Qiagen Multiplex PCR Kit, 0.1 μm of ITSintfor2 and ITS2-reverse (LaJeunesse 2002) and DNase-free water was added to make a total volume of 25 μL. PCR conditions were identical to the conditions used for 454 sequencing. Subsequently, 1 μL of the PCR product was used as template in a 25 μL clade-specific PCR containing 12.5 μL Qiagen Multiplex PCR Kit, DNase-free water, 0.1 μm of degenerated reverse primer (5′- TCWCYTGTCTGACTTCATGC-3′) and 0.1 μm of clade *A* (5′-TGGCACTGGCATGC-3′)-, *B* (5′- ATTGCTGCTTCGCTTTCC-3′)- or *C* (5′- TGCTTAACTTGCCCCAAC-3′)-specific forward primers generating 100, 180 and 210 bp amplicons, respectively. PCR conditions were 94 °C for 15 min followed by 35 cycles of 30 s at 94 °C, 30 s at 53 °C and 30 s at 72 °C and a final step of 10 min at 72 °C. Amplicons were checked on a 1% agarose gel, cloned with Invitrogen TOPO-TA cloning kit and sent for Sanger sequencing to the KAUST BioScience Core Laboratory. CodonCode Aligner (CodonCode Corporation, Centerville, MA) was used to process sequences and annotated via BLASTN using the local *Symbiodinium* ITS2 database.

## Results

### Sequence variation among ITS2 copies in the genomes of Symbiodinium

We applied 454-based amplicon sequencing of the ITS2 rDNA region from *Symbiodinium* to a collection of replicated isoclonal cultures (*n* = 7), pooled cultures (*n* = 2) and environmental samples (*n* = 6) (Table1). A total of 218 475 reads were obtained from pyrosequencing with a median length of 314 bp (mean length = 323.11 ± 32.61 SD). After filtering of sequences, 197 181 reads were retained with an average of 13 145.40 sequences per sample, representing 1487 unique ITS2 sequences (Table1, File S4, Supporting information).

**Table 1:** *Symbiodinium* ITS2 pyrosequencing overview. Depicted are clade types (when cultures were used), the respective animal host and collection site, and DGGE typing results (based on the brightest band). For ITS2 pyrosequencing data, the total number of sequence reads (after filtering) and the number of distinct ITS2 copies for each sample are provided. Sequence reads for each sample are further assigned to clade types detailing number of sequence reads, number of distinct ITS2 copies within clades and clade type of the most abundant ITS2 copy. In cases where the clade type of the most abundant ITS2 copy is different from the database sequence, the percentage identity is indicated. In cases where there are two or more equally abundant ITS2 copies, all clade types are indicated

	Culture or Sample name	Clade type	Animal Host and Collection Site	DGGE typing	ITS2 pyrosequencing		Sequences assigned to clade A			Sequences assigned to clade B			Sequences assigned to clade C		
No. of sequences					No. of distinct ITS2 copies	No. of sequences	No. of distinct ITS2 copies	Clade type of most abundant ITS2 copy	No. of sequences	No. of distinct ITS2 copies	Clade type of most abundant ITS2 copy	No. of sequences	No. of distinct ITS2 copies	Clade type of most abundant ITS2 copy
Isoclonal Cultures	CCMP2467 (1)	A1	*Stylophora pistillata*, Gulf of Aqaba	A1	16 681	331	16 589	309	A1	26	5	B1	66	17	C1
	CCMP2467 (2)	A1	*Stylophora pistillata*, Gulf of Aqaba	A1	8565	241	8537	229	A1	14	4	B1	14	8	C1; C1h
	KB8	A1	*Cassiopea xamachana*, Hawaii	A1	14 894	292	14 882	286	A1	9	4	B1	3	2	C41 (99.65%)
	rt-147 (1)	B1	*Pseudoterogorgia bipinnata*, Jamaica	B1	16 843	225	6	3	A1	16 837	222	B1	0	—	—
	rt-147 (2)	B1	*Pseudoterogorgia bipinnata*, Jamaica	B1	18 989	241	0	—	—	18 989	241	B1	0	—	—
	rt-064	B1	*Cassiopea xamachana*, Jamaica	B1	16 042	175	2	1	A1	16 040	174	B1	0	—	—
	rt-152	C1	*Discosoma sanctithomae*, Jamaica	C1	3116	111	1	1	A1 (99.61%)	11	4	B1	3104	106	C1
Pooled Cultures	CCMP2467:rt-147 (1:1)	A1:B1	Pooled cultures CCMP2467:rt-147 (1:1)	B1	15 234	233	100	12	A1	15 134	221	B1	0	—	—
	CCMP2467: rt-147 (1:3)	A1:B1	Pooled cultures CCMP2467:rt-147 (1:3)	B1	14 915	223	18	7	A1	14 897	216	B1	0	—	—
Field-collected Coral specimens	P.Dog.R3.2	—	*Pocillopora verrucosa*, Doga, Red Sea	A1	22 733	299	22 724	297	A1	7	1	B1	2	1	C1 (99.29%)
	P.Maq.R2.19	—	*P. verrucosa*, Maqna, Red Sea	C1h	3683	142	41	12	A1	30	3	B1	3612	127	C1
	P.Maq.R2.7	—	*P. verrucosa*, Maqna, Red Sea	A1	18 331	247	18 224	230	A1	39	3	B1	68	14	C1
	P.Waj.R1.5	—	*P. verrucosa*, Al Wajh, Red Sea	A1	20 284	297	20 080	282	A1	198	14	B1	6	1	C1h (98.57%)
	P.Waj.D7	—	*P. verrucosa*, Al Wajh, Red Sea	C1c	3579	136	38	13	A1	33	5	B1	3508	118	C1
	A.Af.B6	—	*Acropora hemprichii*, Al Fahal Reef, Red Sea	C41	3292	102	4	3	A1	25	4	B1	3263	95	C41

**Figure 1:**
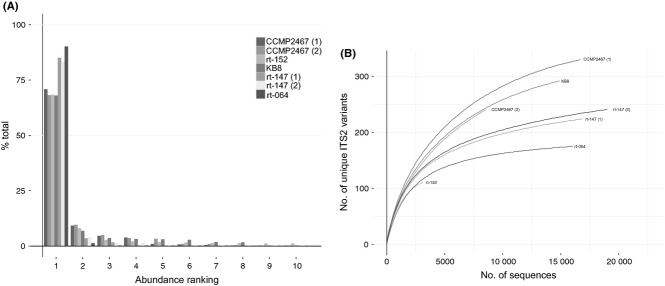
(A) Frequency distribution of sequence reads for the 10 most common ITS2 copies from isoclonal culture samples. (B) Rarefaction curve illustrating ITS2 sequence diversity distribution in isoclonal cultures.

**Figure 2:**
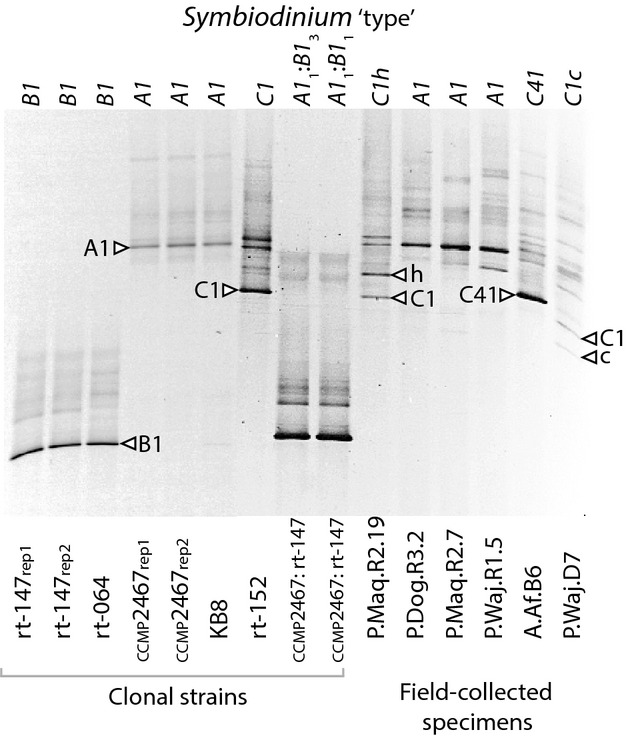
Denaturing gradient gel electrophoresis (DGGE) fingerprint of samples used in this study with clades/subclades indicated.

Depending on the taxon investigated, we identified between 102 and 331 distinct ITS2 sequence variants, including cultured strains and field-collected specimens (mean = 219.67). Taking only isoclonal culture samples into account, we identified on average 230.86 ITS2 sequence variants per culture, indicating that there is a substantial number of distinct ITS2 sequence variants found within *Symbiodinium* genomes (Table1). Despite the high number of distinct ITS2 copies, read counts for the different ITS2 copies showed a highly uneven distribution (Fig.1A). When sorting ITS2 variants of isoclonal cultures by sequence read abundance, the most abundant ITS2 copies were on average ∼20 times more prevalent than the second most common ITS2 copy (all clades: 21.84-fold, clade *A*'s: 8.25-fold, clade *B*'s: 39.88-fold, clade *C*'s: 8.49-fold). Further, the five most abundant ITS2 copies from any culture made up >80% of associated reads, indicating that only few distinct ITS2 genes make up the majority of genomic gene copies. This was substantiated by a rarefaction analysis, which indicated that most of the numerous ITS2 copies were captured at very low abundance in each genome (Fig.1B). For instance, subsampling of isoclonal cultures to 2000 reads yielded on average less than half of the distinct ITS2 copies we were able to recover taking all sequence reads into account. Additionally, the rarefaction curves at this sampling depth did not approach saturation. This effectively illustrates that intragenomic diversity lies in low-abundant genomic ITS2 copies, which also seems to far exceed what could be captured by ‘traditional’ sequencing methods. Comparison of pyrosequencing data of culture CCMP2467 (type *A1*) with 25 sequences generated by cloning and sequencing showed that both techniques identified the same most dominant sequence, but numerical ranking of the next most common sequences did not particularly match up well between cloning and 454 data (not shown). Comparing pyrosequencing data to DGGE fingerprinting of isoclonal *Symbiodinium* cultures, DGGE yielded a single dominant band accompanied by few, faint, background bands. The dominant ITS2 variants produced by 454 pyrosequencing (numerically abundant) and DGGE (brightest band) were identical in sequence and were representative sequences of the *Symbiodinium* type analysed (Table1, Fig.2). Accordingly, 454 pyrosequencing, cloning and sequencing and DGGE produced uniform results in regard to identifying the ITS2 variants most representative of the genome.

To further assess levels of intragenomic ITS2 diversity, we calculated uncorrected genetic distances between all ITS2 variants of a given isoclonal culture (Table2). For each sample, we only considered ITS2 types represented by at least 100 reads. This was done to avoid diversity inflation by potential contamination, methodological artefacts or ultralow-abundant ITS2 copies. The resulting median genetic distance between ITS2 copies from isoclonal cultures was in all cases below 0.02, and median intragenomic variation ranged from 0.003 to 0.017 (Table2). Therefore, most sequence variants within any culture differed by only one or two nucleotide substitutions from each other.

**Table 2:** Intragenomic diversity of ITS2 genes in isoclonal cultures (only ITS2 copies represented by at least 100 reads were considered). Differences are calculated as uncorrected pairwise distances between aligned DNA sequences. The difference between the minimum and maximum genetic distance of ITS2 variants within cultures can be used to derive a similarity cut-off for an operational taxonomic unit (OTU)-based framework, upon which intragenomic diversity is contained within a given OTU

*Symbiodinium* culture	*Symbiodinium* culture type	Mean uncorrected genetic distance	Median uncorrected genetic distance	Minimum	Maximum	Difference (Max-Min)
CCMP2467 (1)	A1	0.006	0.008	0.004	0.008	0.004
CCMP2467 (2)	A1	0.006	0.008	0.004	0.008	0.004
KB8	A1	0.013	0.008	0.004	0.031	0.027
rt-147 (1)	B1	0.005	0.003	0.003	0.007	0.003
rt-147 (2)	B1	0.017	0.017	0.003	0.031	0.027
rt-064	B1	0.003	0.003	0.003	0.003	0.000
rt-152	C1	0.003	0.003	0.003	0.003	0.000

**Figure 3:**
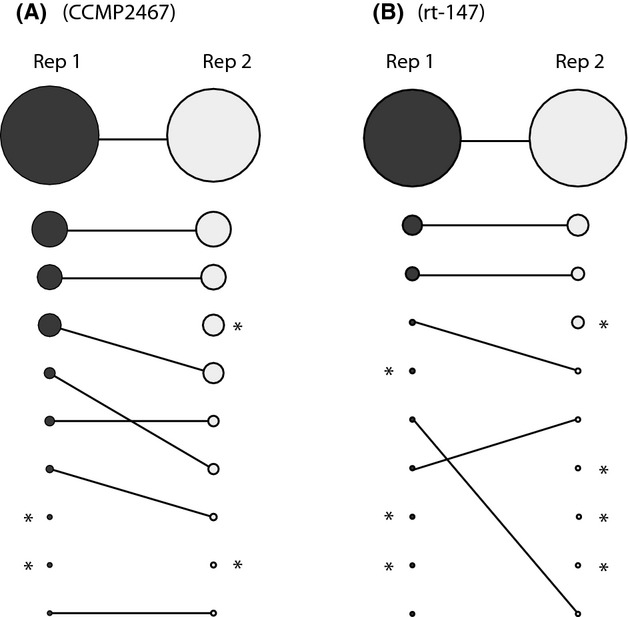
Reproducibility of pyrosequencing rDNA variants for replicated PCRs (using the same DNA extract) of isoclonal cultures (A) CCMP2467 and (B) rt-147 taking the top 10 most abundant sequence variants into account. The sizes of the circles represent relative abundance; circles with an asterisk represent distinct ITS2 variants.

**Figure 4:**
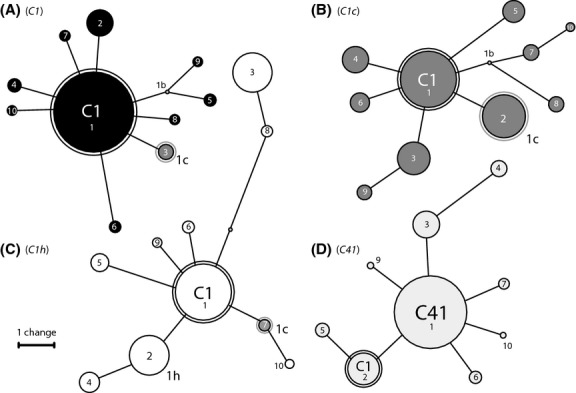
Comparison of top 10 most common variants found from pyrosequencing ITS2 from (A) culture rt-152 (*Symbiodinium goreaui* or type *C1*) and several field-collected samples representing clade *C Symbiodinium*: (B) *C1c* (sample P.Waj.D7), (C) *C1h* (sample P.Maq.R2.19) and (D) C41 (sample A.Af.B6). The size of the circles represents relative abundance, and numbers in circles represent order of distinct ITS2 variants for a given sample. Shared ITS2 variants are depicted in a black outer circle representing ITS2 type *C1*, and a grey outer circle representing ITS2 subtype *c*.

The reproducibility of pyrosequencing rDNA varied depending on the scale of comparison. In some cases, different tallies of distinct ITS2 copies were calculated between the sequencing of different PCR amplifications conducted from the same DNA extract. Our replicated analysis of culture CCMP2467 recovered 331 and 241 distinct ITS2 sequences from 16 681 and 8565 sequence reads, respectively. Of these ITS2 sequences, 184 were identical (55.59 and 76.35%, respectively). Repeated sequencing of the culture rt-147 was less variable and retrieved 225 and 241 distinct ITS2 variants at a comparable sequencing depth, of which 168 were identical (74.67 and 69.71%, respectively) (Table1). The comparison between the top 10 most abundant sequence variants showed consistency, but there were some discrepancies between replicated samples (Fig.3). Sequence variants that represented 2–3% of the total variation were typically recovered between replicates, but not always in the same relative abundance. The top three most common sequences, however, were always recovered, and in the same order of abundance between replicates (Fig.3). To further elucidate this pattern, the top 10 most common variants from pyrosequencing ITS2 of culture rt-152 (*Symbiodinium goreaui* or type *C1*) were compared to the composition of top 10 variants identified from several field-collected samples representing clade *C Symbiodinium*, including types *C1c*, *C1h* and *C41* (Fig.4). For each of these samples, there were one to five common sequence variants representing >5% of sequence reads for the respective specimen. Each sequence set contained the *C1* sequence, *sensu* LaJeunesse (2001), but in very different proportions. The *C1* sequence comprised 68.36% of the genome of *S. goreaui* (rt-152 or type *C1*), 34.00% for type *C1c* (P.Waj.D7), 34.08% for type *C1 h* (P.Maq.R2.19) and 11.82% in type *C41* (A.Af.B6). These proportions were similar to the relative band intensities observed from their respective DGGE fingerprint profile (Fig.2). The ‘*c*’ sequence found in codominance with *C1* (19.95% vs. 34.00%) in the ribosomal array of *Symbiodinium* type *C1c* also was detected in two other types (*C1h* and *C1*) at significantly lower concentrations and explains why this sequence variant was not resolved by DGGE fingerprinting (Fig.2).

Among sequences recovered from PCRs of DNA extracted from the artificially mixed cultures, CCMP2467 (*S. microadriaticum* or type *A1*) and rt-147 (type *B1*), in a 1:1 or 1:3 cell ratio, more than 99% of ITS2 reads were identified only as clade *B*. This result was consistent with DGGE profiling conducted on the same samples, which showed only a single prominent band matching that of the fingerprint for culture rt-147. The band diagnostic of type *A1*, *S. microadriaticum*, was absent in both mixed samples (Fig.2). Sequences derived from 454 (most abundant) and DGGE (brightest band) of both pooled samples were identical and identified as type *B1* via BLASTN. These results suggest that clade *B* has either a far greater copy number of rDNA than clade *A*, or PCR bias of rt-147 over CCMP2467. This bias might arise from differences in DNA isolation efficiency or preferential primer amplification as discussed in Thornhill *et al*. (2013). In conclusion, we could not detect both entities in their initially mixed proportions.

Looking further at ITS2 type composition from pyrosequencing reads of isoclonal cultures, we found that besides the majority of sequences being representatives of the underlying ITS2 type, very low-abundant ITS2 types were present that were representatives of other clades (Table1). These variants matched with the dominant sequence variants (or nearly so) of isolates representing other *Symbiodinium* clades also under analysis and occurred between 0.01 and 0.40% of all reads (Table1). To understand the cause of this observation further, we conducted a series of single-cell PCRs on cultures CCMP2467, KB8, rt-064 and rt-147 with primers specific to clades *A*, *B* and *C*. Clade-specific PCRs performed on single *Symbiodinium* cells from these cultures yielded PCR products with the corresponding clade-specific primers and failed to yield amplicons using primers specific to a clade other than the template, with the exception of culture rt-064 (type *B1*). For this culture, we retrieved PCR products in two of seven cases upon amplification with clade *A*-specific primers. From both PCR products, a total of eight clones were sent for sequencing, of which five were identical to the identified clade *B1* in 454 sequences and three were discarded due to reduced quality.

### Taxon-based analysis of Symbiodinium ITS2 diversity in isoclonal cultures

Studies of microbial diversity in an OTU framework based on pyrosequencing of the 16S rRNA gene have revolutionized our understanding of bacterial diversity and distribution (Sogin *et al.* 2006). Similarly, the ITS2 gene is amenable to analysis in a taxon-based framework under the premise of derivation of appropriate cut-offs to denoting clades and species/types. Given the deep divergence between *Symbiodinium* species from different clades (comparable to differences between orders in other dinoflagellates), we had to devise a strategy where taxonomic delineation was conducted on the clade level first and subsequently at the type level.

Clade separation was empirically determined by pairwise similarity calculation of all sequences and subsequent clustering using a similarity cut-off of >0.10. This approach consistently clustered reads into different clades, which was confirmed by BLASTing representatives of each cluster against our custom ITS2 database. As mothur calculates exact distance cut-offs, the next higher distant cut-off from 0.10 was chosen for data analysis (here: 0.15). After aligning, trimming and discarding sequences that were shorter than 90% of the reads in each clade, 197 128 sequences remained, of which 101 239 sequences belonged to clade *A*, 82 273 belonged to clade *B*, and 13 616 sequences belonged to clade *C* (Table3).

**Table 3:** *Symbiodinium* ITS2 OTU-based analysis overview. Depicted are samples used in this study and the designated clade type based on DGGE typing. For ITS2 pyrosequencing data, the total number of sequence reads (after filtering and clade-based alignment) and the number of OTUs at a 97% similarity cut-off for each sample are provided. Distribution of sequence reads over OTUs on a clade basis and the ITS2 clade type most similar to an OTU are detailed. In cases where the OTU is different from the ITS2 clade type database sequence, the percentage identity is indicated

	Culture or Sample name	DGGE typing	ITS2 pyrosequencing		Clade A OTUs			Clade B OTUs		Clade C OTUs
			No. of sequences	No. of OTUs	No. of sequences OTU1_A0.03_	No. of sequences OTU2_A0.03_	No. of sequences OTU3_A0.03_	No. of sequences OTU1_B0.03_	No. of sequences OTU2_B0.03_	No. of sequences OTU1_C0.03_
Isoclonal cultures	CCMP2467 (1)	A1	16 681	3	16 589	0	0	26	0	66
	CCMP2467 (2)	A1	8565	3	8537	0	0	14	0	14
	KB8	A1	14 891	3	14 879	0	0	9	0	3
	rt-147 (1)	B1	16 836	2	6	0	0	16 830	0	0
	rt-147 (2)	B1	18 987	1	0	0	0	18 987	0	0
	rt-064	B1	16 042	2	2	0	0	16 040	0	0
	rt-152	C1	3116	3	1	0	0	11	0	3104
Pooled Cultures	CCMP2467: rt-147 (1:1)	B1	15 231	2	100	0	0	15 131	0	0
	CCMP2467: rt-147 (1:3)	B1	14 911	2	18	0	0	14 893	0	0
Field-collected Coral specimens	P.Dog.R3.2	A1	22 733	4	22 663	61	0	7	0	2
	P.Maq.R2.19	C1h	3653	3	41	0	0	30	0	3582
	P.Maq.R2.7	A1	18 327	6	18 146	70	4	31	8	68
	P.Waj.R1.5	A1	20 284	4	20 080	0	0	143	55	6
	P.Waj.D7	C1c	3579	3	38	0	0	33	0	3508
	A.Af.B6	C41	3292	3	4	0	0	25	0	3263
			**ITS2 clade type**		A1	A1 (97.27%)	A13 (98.04%)	B1	B2	C1

For the determination of species- or type-level cut-offs, we used data from isoclonal cultures. Our aim was to determine a cut-off that effectively clustered all reads from culture samples assorted to a clade (step above) into one corresponding OTU at the ‘species’ level. Average neighbour clustering of reads based on uncorrected pairwise distances at 0.03 provided the cut-off where all cultures collapsed into one OTU for a given clade (Table3). Therefore, 97% sequence similarity was implemented as a cut-off value for species/type-level OTU-based analyses within clades. Comparing results from sequence- (Table1) and OTU-based (Table3) analyses effectively illustrates that the high number of distinct ITS2 copies that we identified in isoclonal culture samples could be collapsed to within-species (i.e. within OTU_0.03_) ITS2 variation in a taxon-based analysis. Three distinct OTUs_0.03_ (1 of each clade *A*, *B* and *C*) represented data from all cultures. For the pooled culture samples, two OTUs_0.03_ were correctly identified, but the relative abundances of reads did not reflect the initial ratio of cells used. Accordingly, while diversity was correctly recovered for cells from pooled isoclonal cultures, the relative abundances were not.

### Taxon-based analysis of ITS2 diversity in environmental samples

We applied our OTU-based framework to analyse ITS2 diversity in environmental samples based on the cut-offs we derived from the isoclonal cultures (File S5, Supporting information). Similar to the results for the isoclonal culture samples, the amount of type-level OTUs_0.03_ was dramatically lower than the number of distinct ITS2 copies. Diversity in environmental samples was comprised of only six OTUs_0.03_ that represented a total of 841 distinct ITS2 sequences, demonstrating that taxon-based framework analyses drastically and effectively reduce complexity and diversity of primary sequence data. Of the six OTUs_0.03_ that we identified across all environmental samples, three OTUs could be assigned to clade *A* (referred to as OTU1_A0.03_, OTU2_A0.03_ and OTU3_A0.03_), two OTUs to clade *B* (referred to as OTU1_B0.03_ and OTU2_B0.03_) and one OTU to clade *C* (referred to as OTU1_C0.03_) (Table3). OTU1_A0.03_, OTU1_B0.03_ and OTU1_C0.03_ were the most common ITS2 types representing >99% of all sequences for a given clade. These OTUs were classified belonging to *Symbiodinium* types *A1*, *B1* and *C1*, each with 100% sequence identity (Table3). Interestingly, all environmental samples were dominated by a single OTU_0.03_. At the same time, all environmental samples comprised more than one OTU, but the fraction of sequence reads representing additional OTUs was low. This diversity was not captured in the DGGE fingerprint. For instance, DGGE fingerprinting of samples P.Dog.R3.2 and P.Maq.R2.7 only showed association with *Symbiodinium A1*. In our pyrosequencing data, although both samples were dominated by OTU1_A0.03_ representing clade *A1* (∼99% of all reads), 0.27 and 0.38% of reads represented OTU2_A0.03_ and 0.02% of all reads from sample P.Maq.R2.7 represented OTU3_A0.03_ (Table3). Similarly, while P.Maq.R2.19 was dominated by OTU1_C0.03_ in pyrosequencing data (98.06% of sequence reads), it was also associated with clade *A1* (OTU1_A0.03_: 1.12%) and clade *B1* (OTU1_B0.03_1: 0.82%). Finally, even though P.Maq.R2.7 was the most diverse sample, it still could be represented by only six OTUs, effectively demonstrating how OTU-based analyses collapse the majority of genetic diversity into intragenomic (i.e. within OTU) variation.

Comparing the OTU-based analysis to DGGE fingerprinting, we found that for the majority of samples, the representative OTUs were identical to the *Symbiodinium* ITS2 type derived from DGGE rDNA fingerprinting when the most prominent band was sequenced. For sample A.Af.B6, however, the most prominent DGGE band was represented by ITS2 type *C41*. While this type was also the most abundant pyrosequencing read in that sample, *C41* collapsed into a single OTU (i.e. OTU1_C0.03_) with the *C1* ITS2 type. Similarly, in some cases DGGE fingerprinting identified ITS2 types that were not readily identified as distinct OTUs from pyrosequencing data. For instance, while P.Maq.R2.19 was primarily associated with clade *C1* ITS2 type, it was also associated with clade *C1h* based on the DGGE profile. Further, sample P.Waj.D.7 showed an association with *C1c* in addition to clade *C1* in the DGGE profile. *C1h* and *C1c* were readily detected in pyrosequencing data, but represented by/collapsed to a single OTU (i.e. OTU1_C0.03_). This can be attributed to a single-base pair difference between *C1*, *C41*, *C1h* and *C1c*. From a taxon-based analysis point of view, intergenomic divergence did not exceed intragenomic divergence and accordingly could not be resolved into distinct OTUs.

## Discussion

### Sequence variation among ITS2 copies in the genomes of Symbiodinium

We applied 454 pyrosequencing to analyse intra- and intergenomic *Symbiodinium* rDNA diversity in order to gain an in-depth perspective on the relative homogeneity of this high multicopy gene (Thornhill *et al.* 2007). By utilizing isoclonal cultures, we further resolved the degree of genomic homogeneity relative to abundance of ITS2 sequence variants across the ribosomal array. In addition, we extended sequencing resolution in comparison with classical cloning-and-sequencing-based approaches, and we were able to empirically derive cut-offs for application of these types of data in a taxon-based framework. Our analysis protocol was then applied to environmental samples to assess overall feasibility of the approach.

A concern regarding utilization of ITS2 genes for estimating *Symbiodinium* diversity is the multicopy nature of ribosomal DNA (LaJeunesse 2002). The genomes of eukaryotes contain one to several numerically dominant sequence variants (Dover 1986; Hillis & Dixon 1991; LaJeunesse & Pinzon 2007). Species lineages of *Symbiodinium* exist that have two or more codominant ITS2 copies that together are diagnostic of the species (Sampayo *et al.* 2009). The rank abundance plot of ITS2 variants from isoclonal samples (Fig.1A) revealed that while there seems to be extensive intragenomic variation in ITS2 sequences, each genome was characterized (in these particular examples) by the presence of one numerically dominant ITS2 sequence. Provided that the process of concerted evolution is correctly understood (Dover 1986), common intragenomic variants persist in the genomes among the individuals of a genetically recombining population over long evolutionary timescales (Sampayo *et al.* 2009; Thornhill *et al.* 2014). The number of ITS2 sequence variants follows a long-tail abundance distribution with dominant variants (often one or two) being present at high frequency and a high number of rare variants at much lower frequencies (Fig.1). This disparity among intragenomic variants indicates that gene conversion drives the relative homogenization of this gene array, but is not rapid enough to dampen out the appearance and partial spread of new mutations. As rare variants are removed or replaced through concerted evolution, variants derived from the dominant sequence are continually being generated. This process, however, leads to evolutionary stability of the dominant sequence variant(s). For this reason, the numerically dominant intragenomic variant is used to conservatively characterize *Symbiodinium* (LaJeunesse 2002; Thornhill *et al.* 2007). Accordingly, DGGE-based analyses focus only on the dominant *Symbiodinium* ITS2 sequence variants in a sample to resolve the most abundant *Symbiodinium* species present in the host. However, DGGE-based analyses are relatively labour- and time intensive, which might prohibit studying a large number of samples. In comparison, pyrosequencing requires little hands-on time and has become comparatively cheap. In addition, the resolution of additional *Symbiodinium* that might occur at low abundances (<5%) may be detected. DGGE can usually detect the coexistence of a second or third *Symbiodinium* if present at >5–10% of the sample (Thornhill *et al.* 2006), with some species (e.g. type *D1* in *Pocillopora*) requiring 10–30% abundance to be detected (LaJeunesse *et al.* 2008). In contrast, pyrosequencing detected sequences below 1% in our data. Accordingly, DGGE-based ITS2 typing can miss the presence of background species that may be ecologically important (LaJeunesse *et al.* 2009).

The application of deep sequencing can be used to better understand intragenomic rDNA diversity and its evolution within a genome. Pyrosequencing repeatedly identified a large number of ITS2 sequence variants diagnostic of a particular genome (Fig.3) beyond the resolution of DGGE- or cloning-based approaches. Still, the median intragenomic sequence divergence was <0.02 (Table2) similar to the values calculated when only the most abundant sequence variants were considered (Sampayo *et al.* 2009; Stat *et al.* 2013), and this value was subsequently used to inform cut-off estimates for the resolution of separate taxa.

The analysis of mixed cultured isolates at different ratios showed that one must be conservative when interpreting abundance in samples with two or more *Symbiodinium* species. This is mainly due to the differences in rDNA copy number between *Symbiodinium* species (Rowan & Knowlton 1995; Rowan *et al.* 1997; Mieog *et al.* 2007; LaJeunesse *et al.* 2008), or differences in DNA extraction efficiency among different *Symbiodinium* spp. Pooled cultures of *Symbiodinium A1* (CCMP2467) and *B1* (rt-147) in a 1:1 ratio generated far more sequence reads for *Symbiodinium B1*, despite pooling the same number of *Symbiodinium* cells from both cultures (Table1). While genomic complexity in terms of distinct copies was similar for both cultures, clade *B* has several times more ITS copies than clade *A*, explaining the unequal read distribution we recovered (T. C. LaJeunesse, unpublished data). Accordingly, this has to be taken into account when using rDNA in estimating the relative abundances of samples containing members of two or more clades.

Our discovery of ITS2 sequence variants diagnostic of a different clade in pyrosequencing data from isoclonal cultures prompted further investigations via single-cell PCRs. Given the absence of consistent foreign clade amplification in single-cell PCRs as well as the vast evolutionary timescales between *Symbiodinium* clades (estimated at tens of millions of years), the most parsimonious explanation is DNA contamination (e.g. in the form of aerosols). In a recent review, van Oppen & Gates (2006) report on the retrieval of highly divergent sequences (diagnostic of other clade entities) at ultralow abundance, indicating that contamination is potentially a significant issue in laboratories that extensively employ PCRs of the ITS2 marker. The finding of unexpected ‘background’ sequences would need to be confirmed by next-generation sequencing these samples in laboratories that have not previously worked on *Symbiodinium* ITS2.

### Taxon-based analysis of ITS2 diversity

While many studies have applied cluster-based approaches to analyse *Symbiodinium* ITS2 diversity within environmental samples (Correa & Baker 2009; Stat *et al.* 2013), few studies yet have targeted analysing *Symbiodinium* diversity with high-throughput sequencing data in an OTU-based framework (Green *et al.* 2014; Thomas *et al.* 2014). We think that this is the most suitable approach in dealing with data produced from next-generation sequencing of rDNA. It should be noted that the application of high-throughput sequencing of the ITS2 region is not limited to the Roche 454 platform. Given current read-length improvements of the Illumina MiSeq platform (2*300 bp Paired-End libraries) in addition to its higher throughput, data from this platform can readily be applied to the analysis of ITS2 in a taxon-based framework. In particular, our OTU-based pipeline developed here for mothur is compatible and portable to data generated on the Illumina MiSeq platform with minor changes to the quality trimming steps (Phase I, File 2, Supporting information) based on differences in the sff (Roche 454) and fastq (Illumina MiSeq) file format. The challenge lies in the derivation of appropriate cut-offs for the separation of sequences into clades and species or types. Even for a well-studied marker such as 16S, taxonomic cut-offs are not necessarily accurate (Ghyselinck *et al.* 2013; Preheim *et al.* 2013). Here, we used (replicated) sequencing of isoclonal cultures representing clades *A*, *B* and *C* to better understand inter- and intragenomic diversity and empirically derive taxonomic cut-offs. Future studies should sequence rDNA from additional cultures representing a diverse set of *Symbiodinium* species.

On the clade level, due to the large evolutionary distance in the genus *Symbiodinium*, ITS2 sequences from different clades are difficult if not impossible to align correctly. Accordingly, taxon-based framework analyses will fail to separate ITS2 sequences into ecological and evolutionarily discrete entities when sequences from all clades are considered at the same time. Rather, it will lead to exaggerated genetic distance estimates retrieved from the alignment of nonhomologous DNA characters. To resolve ITS2 types more accurately, we found that sequences need to be separated into distinct clades first, and a subsequent OTU-based analysis has to be carried out for each clade separately. For our data, ITS2 sequences separated into clades upon applying a cut-off value of 0.15. However, application of this cut-off to ITS2 sequences from our database considering all clades (i.e. A–I) did not correctly cluster ITS2 sequences into all distinct clades (data not shown). This might be due to difficulties in calculating pairwise similarities between sequences when all subtypes of *Symbiodinium*, for example A1, A2, A3 or B1, B2, B3 are present in one data set. To separate ITS2 sequences into *Symbiodinium* clade levels, we suggest manual binning upon determination of an appropriate similarity cut-off before conducting OTU-based ITS2 type analyses (File S5, Supporting information).

Similarly as for the separation of ITS2 reads into clades, there is no *a priori* cut-off value that consistently sorts ITS2 sequences into distinct species (Thornhill *et al.* 2014). In fact, *Symbiodinium* species classification should not be conducted using ITS2 sequence divergence alone, as ITS2 may not consistently resolve species. For instance, two distinct *Symbiodinium* lineages that are not undergoing genetic exchange can have the same ITS2 sequence (Thornhill *et al.* 2014). Accordingly, a taxonomic cut-off value that clusters existent ITS2 variants into OTUs should be regarded as provisional, pending subsequent analysis with independent and more rapidly evolving markers. For this reason, grouping of ITS2 variants into provisional OTUs for the (initial) characterization and comparison of *Symbiodinium* diversity seems a more feasible approach than trying to definitely resolve ITS2 sequence differences into species.

As ITS2 rDNA is not a single-copy gene, a cut-off value that clusters intragenomic ITS2 variants into a single OTU might be indicated as a valid cut-off value. In our data, clustering sequences from isoclonal cultures assorted to clades with the average neighbour algorithm (Schloss & Westcott 2011) at a pairwise genetic distance cut-off of 0.03 collapsed intragenomic ITS2 variation into a single OTU. For this reason, we adopted 0.03 as a cut-off to determine *Symbiodinium* community composition from pyrosequencing data retrieved from field-collected coral specimens. The application of this cut-off to samples from coral host tissues resulted in only six distinct OTUs describing *Symbiodinium* diversity, despite numerous sequenced ITS2 variants. This provides an indication of how much of the variation we sequenced might be attributable to actual intra- rather than intergenomic diversity. Applying an OTU-based approach to the analysis of coral symbiont diversity suggests that most coral colonies appear to be dominated by a single genetic entity (Sampayo *et al.* 2009; Pettay *et al.* 2011; Baums *et al.* 2014; Thornhill *et al.* 2014). However, ITS2 sequences of types *C1*, *C1h*, *C1c* and *C41* were represented by the same OTU at a 0.03 cut-off underscoring the difficulties that are associated with matching ITS2 sequence variants to closely related *Symbiodinium* with separate ecological distributions. Because these entities share a recent common ancestor as members of the *C1* radiation (*sensu* Thornhill *et al.* 2014), the genomes of these *Symbiodinium* shared the ancestral sequence, *C1*, at different relative abundances (Fig.4). An exception was a *Pocillopora verrucosa* specimen (P.Maq.R2.7, Table3): in this sample, we detected all six OTUs representing the entire OTU diversity from our data. The significance of colonies possibly harbouring highly mixed *Symbiodinium* assemblages is not fully understood and should be the effort of future studies.

### Considerations for the application of an OTU-based pipeline to the analysis of Symbiodinium ITS2 diversity

There is probably no single genetic marker that can correctly classify all *Symbiodinium* diversity into distinct species (LaJeunesse *et al.* 2012; Pochon *et al.* 2012). Typically, a distinct ITS2 variant that is numerically dominant in the genome is tentatively regarded as a new type, or ‘species’, of *Symbiodinium* (LaJeunesse 2002; LaJeunesse *et al.* 2010), and only when diagnostic of an ecologically distinct population. However, for (initial) characterization of *Symbiodinium* diversity in a large number of samples, elucidation of *Symbiodinium* species using multimarker molecular evidence in combination with morphological, ecological and physiological data (as detailed in, e.g., LaJeunesse *et al.* 2012) is not (yet) feasible. Here, we suggest adoption of an OTU-based framework to analyse high-throughput *Symbiodinium* ITS2 sequence data to provisionally assess species diversity (similar to the use of DGGE fingerprinting of ITS2 rDNA) until ‘true’ species are formally described using the convergence of independent lines of genetic, ecological and in some cases morphological evidence (LaJeunesse *et al.* 2014). Although the application of a taxon-based framework will miss some ecologically and evolutionarily distinct entities, most importantly, it provides a conservative and comparable estimate of *Symbiodinium* diversity based on rDNA. The development of sensible cut-offs to delineate *Symbiodinium* diversity in an OTU-based framework allows harmonization and reanalysis of existing and new data under a common set of rules. We specifically acknowledge that some *Symbiodinium* species will be contained in a single OTU and not resolve at a cut-off of >97% similarity. For instance, clade *C*, which is by far the most diverse clade with a broad radiation, would require higher cut-off values to resolve species. But as ITS2 data alone are unable to unequivocally diagnose species, our intentions were to provide a reasonable cut-off that investigators can apply to compare diversity within and between their samples and that deflates rather than inflates *Symbiodinium* species diversity estimates. Further, continuing efforts in the (genetic) description of *Symbiodinium* species (LaJeunesse *et al.* 2012, 2014; Jeong *et al.* 2014) and the ease of typing a large number of samples might encourage the use of multiple markers in the analysis of *Symbiodinium* diversity, which will facilitate analysis of high-throughput sequencing data in a phylogeny-based context. This perspective stimulates further comparative analyses of ocean basins and species in a standardized framework that might provide greater insight into symbiont diversity of marine invertebrates and the acclimation of corals to environmental change.

## Data accessibility

ITS2 DNA variants were uploaded as online supplemental material. Pyrosequencing analysis pipeline overview and mothur script were uploaded as online supplemental material. The sequences determined in this study have been deposited in the NCBI Sequence Read Archive under accession no. PRJNA244965.

## Supporting Information

File S1 ITS2 BLAST database.

File S2 *Symbiodinium* ITS2 analysis pipeline with mothur (version 1.31.2).

File S3 Script file for unattended mothur ITS2 analysis.

File S4 Overview over ITS2 classification, read counts, and reference sequence for all ITS2 variants identified.

File S5 Overview over ITS2 *Symbiodinium* 454 Data Analysis Pipeline.
