# Supplementary material for: Assessing Symbiodinium diversity in scleractinian corals via next-generation sequencing-based genotyping of the ITS2 rDNA region
Source: Mol Ecol. 2014 Aug 18;23(17):4418–33. doi: 10.1111/mec.12869 (PMC4285332; doi:10.1111/mec.12869)
Supplement: File S2 — Symbiodinium ITS2 analysis pipeline with mothur (version 1.31.2). [file mec0023-4418-SD2.docx]

***Symbiodinium* ITS2 Analysis Pipeline with mothur (version 1.31.2), Voolstra lab, April 2014**

*Note: the main difference in comparison to OTU-based analyses with 16S is that ITS2 sequences from different clades cannot be aligned properly; for this reason sequences must be separated by clade after general sequence trimming and filtering*

**Phase I: Quality trimming**

**1. Extract Fasta, Qual, Flow files from .sff file**

mothur > sffinfo(sff=H2SYBJJ03.sff, flow=T)

mothur > summary.seqs(fasta=H2SYBJJ03.fasta)

**2. Sort out bad seqs from the flowgram (need oligos file), split seqs by barcodes (flow file)**

mothur > trim.flows(flow=H2SYBJJ03.flow, oligos=oligos.oligos, bdiffs=0, pdiffs=2, minflows=250, maxflows=800, fasta=T, processors=4)

**3. Denoise the flowgrams with the PyroNoise Algorithm**

# LookUp_Titanium.pat file needed; download from mothur wiki

mothur > shhh.flows(file=H2SYBJJ03.flow.files, processors=4)

- H2SYBJJ03.shhh.fasta, H2SYBJJ03.shhh.names

**4. Trim sequences according to parameters**

*# 2 errors in primer, 0 errors in barcode; discard any seqs ≥ 1 ambiguous bp; discard seqs < 250bp*

*# oligo file holds barcode and forward sequencing primer sequence*

mothur > trim.seqs(fasta=H2SYBJJ03.shhh.fasta, name=H2SYBJJ03.shhh.names, oligos=oligos.oligos, pdiffs=2, bdiffs=0, maxambig=0, maxhomop=4, minlength=250, processors=4, allfiles=T)

- H2SYBJJ03.shhh.scrap.fasta

*# Check why seqs are being discarded from trim.seqs in the scrap.fasta file*

bash$ cut -d ' ' -f 1 H2SYBJJ03.shhh.scrap.fasta | perl -F'\|' -anle '$h{ $F[1] }++; END{print "$_\t$h{$_}" for keys %h}' > H2SYBJJ03.shhh.scrap.fasta.count

*# Check summary.seqs to determine number of seqs kept*

mothur > summary.seqs(fasta=H2SYBJJ03.shhh.trim.fasta, name=H2SYBJJ03.shhh.trim.names)

*# If many sequences are lost, it might be sensible to increase maxhomop*

**5. Trim reverse primer using cutadapt version 1.1**

*# Allow error rate = 0.15 to allow 2-3 indel/mismatches; reverse primer seq should be in revcomp direction*

bash$ cutadapt -a ACCCGCTGAACTTAAGCATATGGATCCC -e 0.15 H2SYBJJ03.shhh.trim.fasta > H2SYBJJ03.shhh.trim.revtrim.fasta

**6. Collapse identical seqs**

*# Only the representative seqs are kept to reduce computation time*

*# counts of actual number of seqs are retained*

mothur > unique.seqs(fasta=H2SYBJJ03.shhh.trim.revtrim.fasta, name=H2SYBJJ03.shhh.trim.names)

mothur > summary.seqs(fasta=H2SYBJJ03.shhh.trim.revtrim.unique.fasta, name=H2SYBJJ03.shhh.trim.revtrim.names)

mothur > count.seqs(name=H2SYBJJ03.shhh.trim.revtrim.names, group=H2SYBJJ03.shhh.groups)

**7. Check for Chimeras using UCHIME** **and remove them**

*# (http://drive5.com/usearch/manual/uchime_algo.html)*

mothur > chimera.uchime(fasta=H2SYBJJ03.shhh.trim.revtrim.unique.fasta, name=H2SYBJJ03.shhh.trim.revtrim.names, group=H2SYBJJ03.shhh.groups, processors=4)

mothur > remove.seqs(accnos=H2SYBJJ03.shhh.trim.revtrim.unique.uchime.accnos, fasta=H2SYBJJ03.shhh.trim.revtrim.unique.fasta, name=H2SYBJJ03.shhh.trim.revtrim.names, group=H2SYBJJ03.shhh.groups)

mothur > summary.seqs(name=H2SYBJJ03.shhh.trim.revtrim.pick.names)

**8. Remove singletons in mothur**

*# Seqs detected only once across the entire dataset are not included in further analyses*

mothur > split.abund(cutoff=1, fasta=H2SYBJJ03.shhh.trim.revtrim.unique.pick.fasta, name=H2SYBJJ03.shhh.trim.revtrim.pick.names, group=H2SYBJJ03.shhh.pick.groups)

# *Keep record of sequences kept/lost*

mothur > summary.seqs(fasta=H2SYBJJ03.shhh.trim.revtrim.unique.pick.abund.fasta, name=H2SYBJJ03.shhh.trim.revtrim.pick.abund.names)

*# count.seqs for assessing frequency distribution of ITS2 copies*

mothur > count.seqs(name=H2SYBJJ03.shhh.trim.revtrim.pick.abund.names, group=H2SYBJJ03.shhh.pick.abund.groups)

- H2SYBJJ03.shhh.trim.revtrim.pick.abund.count.table (Supplement file 4)

*# Sanity check: align the most abundant seqs in all samples to their respective DGGE seqs to check if they are identical*

# *Copy to files with short names, its2.fasta, its2.names and its2.groups*

bash$ cp H2SYBJJ03.shhh.trim.revtrim.unique.pick.abund.fasta its2.fasta bash$ cp H2SYBJJ03.shhh.trim.revtrim.pick.abund.names its2.names bash$ cp H2SYBJJ03.shhh.pick.abund.groups its2.groups

**Phase II: Clades separation**

**9. Cluster Sequences according to clades and assess divergence cutoffs for clades**

# *First, calculate pairwise similarity between seqs using pairwise.seqs command*

*# Then cluster based on similarity scores (which pairwise.seqs command gives out) using average neighbor option*

mothur > pairwise.seqs(fasta=its2.fasta, calc=onegap, countends=F, processors=4)

mothur > cluster(column=its2.dist, name=its2.names, method=average)

mothur > make.shared(list=its2.an.list, group=its2.groups)

- its2.an.shared *(provides size and number of OTUs over samples for given cutoff)*

**10. Assign OTUs to clades**

*# mothur calculates exact distant cutoffs and provides only results from those cutoffs. Therefore, we have to check seqs in each cutoff and choose a cutoff that separate clusters into different clades (usually it should be > 0.10); caution: use next higher cutoff value as mothur per default selects previous lower one*

# *Get representative sequence for each OTU (i.e. clade level at > 0.10)*

mothur > get.oturep(column=its2.dist, name=its2.names, fasta=its2.fasta, list=its2.an.list, group=its2.groups, label=0.15)

# *Find out which OTU represents which clade via BLASTn (in this data set: 3 sequences)*

mothur > system(blastn -db ~/Databases/ITS2KAUST -query its2.an.0.15.rep.fasta -out 0.15.blast -outfmt 6 -max_target_seqs 1)

# Display BLAST outputs

mothur > system(cat 0.15.blast)

# *In this example: at 0.15 cutoff, there are 3 clusters and they belong to clade A, B and C therefore, this is the cutoff to choose. CHECK via BLASTn that the OTUs generated belong to different clades*

**11. Generate a fasta file of all sequences associated to OTUs at the clade level**

mothur > bin.seqs(name=its2.names, fasta=its2.fasta, list=its2.an.list, label=0.15)

- its2.an.0.15.fasta (this is the complete 454 data sorted according to clade level)

**12. Separate sequences by clade-level OTUs to different fasta files**

# *Save files as its2.A.fasta, its2.B.fasta and its2.C.fasta*

# *For example, using grep*

bash$ grep -A 1 -P '>.+\t1$' its2.an.0.15.fasta > its2.A.fasta

bash$ grep -A 1 -P '>.+\t2$' its2.an.0.15.fasta > its2.B.fasta

basj$ grep -A 1 -P '>.+\t3$' its2.an.0.15.fasta > its2.C.fasta

**13. Sanity check: BLAST fasta file from each clade to check that all sequences match the same clade.**

bash$ blastn -db ~/Databases/ITS2KAUST -query its2.A.fasta -out its2.A.blast -outfmt 6 -max_target_seqs 1

bash$ blastn -db ~/Databases/ITS2KAUST -query its2.B.fasta -out its2.B.blast -outfmt 6 -max_target_seqs 1

bash$ blastn -db ~/Databases/ITS2KAUST -query its2.C.fasta -out its2.C.blast -outfmt 6 -max_target_seqs 1

**14. Collapse to unique seqs per clade to reduce computation time**

# *Do for each clade identified*

mother > unique.seqs(fasta=its2.A.fasta)

mother > summary.seqs(fasta=its2.A.unique.fasta, name=its2.A.names)

**15. Align unique seqs of each clade with MUSCLE**

# *Do for each clade identified*

mothur > system(muscle -in its2.A.unique.fasta -out its2.A.unique.align)

**16. Trim seqs from each clade to equal length**

# *The format of aligned fasta files need to be modified since* *MUSCLE does not put “.” at the beginning or end of alignments, and outputs block fasta format. The following command changes that*

# *Do for each clade identified*

bash$ perl -ne 'if ($. == 1) {print; next}; chomp; if (/^>/) { print "\n", $_, "\n"} else { print }' its2.A.unique.align | perl -pe 's/^(-+)/"." x length($1)/eg; s/(-+)$/"." x length($1)/eg;' > its2.A.unique.dots.align

mothur > summary.seqs(fasta=its2.A.unique.dots.align, name=its2.A.names)

# *The following needs to be adjusted for the actual alignment; choose start and end so that a majority of the sequences can be retained (here: optimize option = 90 was used in the screen.seqs, which automatically trim to keep 90% of the sequences)*

mothur > screen.seqs(fasta=its2.A.unique.dots.align, optimize=start-end, criteria=90, name=its2.A.names)

mothur > summary.seqs(fasta=its2.A.unique.dots.good.align, name=its2.A.names)

*# The filter.seqs command will cut off ‘overhangs’ at both ends of the alignment, and remove any columns that are all gaps*

mothur > filter.seqs(fasta=its2.A.unique.dots.good.align, trump=., vertical=T)

mothur > summary.seqs(fasta=its2.A.unique.dots.good.filter.fasta, name=its2.A.good.names)

**17. De-collapse unique seqs for each clade to get all seqs, save to fasta file**

# *Do for each clade identified*

mothur > deunique.seqs(fasta=its2.A.unique.dots.good.filter.fasta, name=its2.A.good.names)

- its2.A.redundant.fasta

**18. Extract seq IDs and sample names from each clade's fasta file for use as a seq ID file and a group file in mothur**

*# to continue analysis in mothur group files are needed for each clade*

*# Save as its2.all.A.seqIDs and its2.all.A.groups, accordingly for all clades identified*

*# Do for each clade identified*

bash$ grep \> its2.A.redundant.fasta | perl -pe 's/>//'> its2.all.A.seqIDs

*# Get the group information from main group file, write to new clade group file.*

bash$ perl -ne 'BEGIN{open $IN, "<", "its2.groups"; while (<$IN>){chomp; $db{( split /\t/ )[0]}=$_;}} chomp; if ( exists $db{$_} ) { print "$db{$_}\n" }' its2.all.A.seqIDs > its2.all.A.groups

**Phase III: ITS2 type separation**

*# It is recommended to do the following steps in separate directories per clade*

*# Start the community analysis for each clade separately but over all samples*

**19. Use the outputs from step 18. Collapse identical sequences to reduce computation time**

*# Do for each clade identified*

mothur > unique.seqs(fasta=its2.A.redundant.fasta)

**20. Calculate uncorrected pairwise distance and cluster seqs into OTUs at a 0.03 cutoff**

*# Do for each clade identified*

mothur > dist.seqs(fasta=its2.A.redundant.unique.fasta)

mothur > cluster(column=its2.A.redundant.unique.dist, name=its2.A.redundant.names, method=average)

mothur > make.shared(list=its2.A.redundant.unique.an.list, group=its2.all.A.groups)

- its2.A.redundant.unique.an.shared (provides size and number of OTUs over all samples)

**21. Get a representative sequence (the most abundant sequence) for each OTU at a 0.03 cutoff and annotate with local database (BLASTn)**

*# Do for each clade identified*

mothur > get.oturep(fasta=its2.A.redundant.unique.fasta, list=its2.A.redundant.unique.an.list, name=its2.A.redundant.names, label=0.03, method=abundance)

bash$ blastn -db ~/Databases/ITS2KAUST -query its2.A.redundant.unique.an.0.03.rep.fasta -out its2.A.0.03rep.blast -outfmt 6 -max_target_seqs 3

**22. Get ITS2 sequence distribution over all samples for any given clade (optional)**

*# Do for each clade identified*

mothur > count.seqs(name=its2.A.redundant.names, group=its2.all.A.groups)
